# Nirmatrelvir-Ritonavir and COVID-19 Mortality and Hospitalization Among Patients With Vulnerability to COVID-19 Complications

**DOI:** 10.1001/jamanetworkopen.2023.36678

**Published:** 2023-10-02

**Authors:** Colin R. Dormuth, Jason D. Kim, Anat Fisher, Jolanta Piszczek, I Fan Kuo

**Affiliations:** 1 Department of Anesthesiology, Pharmacology and Therapeutics, University of British Columbia, Vancouver, British Columbia, Canada; 2 Therapeutics Initiative, University of British Columbia, Vancouver, British Columbia, Canada; 3 Department of Pharmaceutical Sciences, University of British Columbia, Vancouver, British Columbia, Canada; 4 BC COVID Therapeutics Committee, Vancouver, British Columbia, Canada; 5 Pharmaceutical Laboratory and Blood Services Division, British Columbia Ministry of Health, Vancouver, British Columbia, Canada

## Abstract

**Importance:**

Postmarket analysis of individuals who receive nirmatrelvir and ritonavir (Paxlovid [Pfizer]) is essential because they differ substantially from individuals included in published clinical trials.

**Objective:**

To examine the association of nirmatrelvir and ritonavir with prevention of death or admission to hospital in individuals with different risks of complications from COVID-19 infection.

**Design, Setting, and Participants:**

This is a cohort study of adult patients in British Columbia, Canada, between February 1, 2022, and February 3, 2023. Patients were eligible if they belonged to 1 of 4 higher-risk groups of individuals who received priority for COVID-19 vaccination. Two groups included clinically extremely vulnerable (CEV) people who were severely (CEV1) or moderately immunocompromised (CEV2). CEV3 individuals were not immunocompromised but had medical conditions associated with a high risk for complications from COVID-19. A fourth expanded eligibility (EXEL) group was added to allow wider access to nirmatrelvir and ritonavir for certain other higher-risk individuals who were not in a CEV group, such as those older than 70 years who were unvaccinated.

**Exposures:**

Patients with COVID-19 who received nirmatrelvir and ritonavir were matched to patients in the same vulnerability group; who were of the same sex, age, and propensity score for nirmatrelvir and ritonavir treatment; and who were also infected within 1 month of the individual treated with nirmatrelvir and ritonavir.

**Main Outcomes and Measures:**

The primary outcome was death from any cause or emergency hospitalization with COVID-19 within 28 days.

**Results:**

There were 6866 individuals included in the study, of whom 3888 (56.6%) were female and whose median (IQR) age was 70 (57-80) years. Compared with unexposed controls, treatment with nirmatrelvir and ritonavir was associated with statistically significant relative reductions in the primary outcome in the CEV1 group (560 patients; risk difference [RD], −2.5%, 95% CI, −4.8% to −0.2%) and the CEV2 group (2628 patients; RD, −1.7%; 95% CI, −2.9% to −0.5%). In the CEV3 group, the RD was −1.3%, but the findings were not statistically significant (2100 patients; 95% CI, −2.8% to 0.1%). In the EXEL group, treatment was associated with higher risk of the outcome (RD, 1.0%), but the findings were not statistically significant (1578 patients; 95% CI, −0.9% to 2.9%).

**Conclusions and Relevance:**

In this cohort study of 6866 individuals in British Columbia, nirmatrelvir and ritonavir treatment was associated with reduced risk of COVID-19 hospitalization or death in CEV individuals, with the greatest benefit observed in severely immunocompromised individuals. No reduction in the primary outcome was observed in lower-risk individuals, including those aged 70 years or older without serious comorbidities.

## Key Points

**Question:**

What is the association of nirmatrelvir and ritonavir exposure with the risk of death or COVID-19–related hospitalization when accounting for patient vulnerability to complications from COVID-19 infection?

**Findings:**

In this cohort study of 6866 individuals with COVID-19, treatment with nirmatrelvir and ritonavir was associated with lower risk of death or hospitalization in the most clinically extremely vulnerable individuals but not in less vulnerable individuals. Individuals who were not extremely vulnerable to experiencing complications from COVID-19, whose median age was 79 years, had greater risk of the outcome while receiving nirmatrelvir and ritonavir, but the finding was not statistically significant.

**Meaning:**

In this study, treatment with nirmatrelvir and ritonavir was not associated with reduced risk of death or hospitalization among individuals who were not extremely vulnerable to complications from COVID-19 infection, regardless of age.

## Teaser

This cohort study examines the association of nirmatrelvir and ritonavir with prevention of death or admission to hospital in individuals with different risks of complications from COVID-19 infection.

## Introduction

Nirmatrelvir and ritonavir (Paxlovid [Pfizer]) is an oral antiviral drug combination that targets a key SARS-CoV-2 protease enzyme. Nirmatrelvir and ritonavir was approved based on interim efficacy and safety data from the Evaluation of Inhibition for COVID-19 in High-Risk Patients (EPIC-HR) trial, conducted before the emergence of the Omicron variant.^1,2^ In the trial, 2246 patients who were not vaccinated against COVID-19 were randomized to receive nirmatrelvir and ritonavir or placebo within 5 days of symptom onset. Nirmatrelvir and ritonavir reduced the primary composite end point of 28-day risk of death or COVID-19–related hospitalization by 5.6% absolute (88% relative) compared with placebo. By day 28, there were 0 deaths in the nirmatrelvir and ritonavir group and 12 in the placebo group. EPIC-HR reported numerically fewer serious adverse events but more suspected drug-related adverse events with nirmatrelvir and ritonavir than placebo.

Parallel to EPIC-HR, a further 1141 lower-risk adults were studied in a second trial known as EPIC-SR (called standard-risk).^3,4^ The manufacturer announced the closure of the trial in a June 14, 2022, media release, “due to a very low rate of hospitalization or death observed in the standard-risk patient population.”^4^ Nirmatrelvir and ritonavir was not associated with reduced symptoms (the primary end point), and the media release reported a non–statistically significant reduction of 0.9% absolute (51% relative) in hospitalization or death. Peer-reviewed observational analysis has also shown COVID-19–related hospitalization or death from any cause was significantly lower with nirmatrelvir and ritonavir treatment,^5^ although by approximately half the magnitude reported in the EPIC-HR trial. Observational studies have demonstrated a benefit of nirmatrelvir and ritonavir treatment in higher risk individuals.^5,6^

Protective associations with nirmatrelvir and ritonavir therapy have been shown to be variable across the different populations and study periods, which suggests the benefit risk profile of nirmatrelvir and ritonavir depends on vulnerability to complications from COVID-19 infection. The benefit-harm profile of nirmatrelvir and ritonavir thus remains uncertain. British Columbia (BC) provides a natural experiment for examining nirmatrelvir and ritonavir according to vulnerability to complications from COVID-19. BC adopted eligibility criteria for nirmatrelvir and ritonavir that differed substantially from participants in the EPIC-HR trial and included individuals who were more comparable with those studied in the EPIC-SR trial. Furthermore, patients enrolled in EPIC-HR were unvaccinated, had no natural immunity from prior COVID-19 infection, were infected by COVID-19 variants that were different from those now circulating, and were not taking drugs with known CYP 3A4 interactions.^7^ As in the EPIC-HR trial, we sought to analyze the 28-day risk of death or COVID-19–associated hospitalization in the 4 groups of vulnerable individuals in BC with elevated risk of complications who were given access to nirmatrelvir and ritonavir.

## Methods

### Study Design and Data Source

We undertook a retrospective cohort study between February 1, 2022, and February 3, 2023, of individuals who had increased vulnerability to complications from COVID-19 infection. Death from any cause and COVID-19–related hospitalization were compared between individuals who were either prescribed or not prescribed nirmatrelvir and ritonavir. The study used anonymized, individual-level, and linkable administrative health databases from the BC Ministry of Health. Prescription data for nirmatrelvir and ritonavir and other drugs were obtained from the PharmaNet database of all prescriptions filled at community pharmacies. COVID-19 vaccination records and polymerase chain reaction (PCR) test records were also obtained from the Ministry of Health. Demographic, diagnostic, and medical procedure data, used to identify clinically extremely vulnerable (CEV) cohorts and perform statistical adjustment, were obtained from the Medical Services Plan database, the hospital Discharge Abstract Database, and the National Ambulatory Care Reporting System database (NACRS). This study followed the Strengthening the Reporting of Observational Studies in Epidemiology (STROBE) reporting guideline. Informed consent from study participants was not required because all data were deidentified and the study satisfied the minimal risk category for ethics review at the University of British Columbia.

### Identification of Study Cohorts

Four mutually exclusive populations of individuals at higher risk for complications from COVID-19 infection were eligible for nirmatrelvir and ritonavir during the study period. Detailed definitions used to identify these populations are provided in eAppendix 1 in Supplement 1. Briefly, there were 4 populations that included individuals with medical conditions that were designated by a group of BC specialists for the purpose of prioritizing COVID-19 vaccinations and treatments.^8^ These groups contained 3 CEV populations (CEV 1, 2 and 3), and 1 expanded eligibility (EXEL) population. Two CEV populations included individuals 18 years of age and older who were either severely (CEV1) or moderately (CEV2) immunocompromised. Individuals in the CEV3 population were not immunocompromised but had medical conditions thought to place them at higher than typical risk for complications from COVID-19 infection. These conditions included severe respiratory disorders; certain blood disorders, metabolic disorders, and cancers not captured in the other CEV groups; or insulin-dependent diabetes. A fourth group, the EXEL population, was added on March 17, 2022, to allow wider access to nirmatrelvir and ritonavir for individuals at lower risk of COVID-19–related complications than CEV patients, but who had risk factors that put them at higher risk of complications compared with the general population. Eligibility details for this category are publicly available from the BC Centre for Disease Control.^8^ Briefly, the EXEL category was focused on older individuals with certain comorbidities who were unvaccinated or undervaccinated according to the Strong Recommendations by the National Advisory Committee on Immunization.^9^ Individuals 70 years of age or older who were unvaccinated could qualify for nirmatrelvir and ritonavir under the EXEL category. Individuals 70 years of age or older who were fully vaccinated but had a serious medical condition not captured in a CEV category could also qualify. Prescribing physicians were allowed to use discretion in their determination of serious comorbidities.

Cohorts of individuals with COVID-19, identified from either a PCR test, a prescription for nrirmatrelvir and ritonavir, or both, were assembled from the 4 vulnerable populations. Individuals were not allowed to enter a cohort more than once. For individuals who at different times qualified as nirmatrelvir and ritonavir exposed and unexposed, the nirmatrelvir and ritonavir instance was included. Prior to initiating treatment with nirmatrelvir and ritonavir or having a positive COVID-19 test, individuals required at least 730 days of continuous enrollment in the BC Medical Services Plan (gaps of ≤30 days were permitted). Individuals were excluded from the study if, at the time before their test sample collection date (or 3 days prior to initiating nirmatrelvir and ritonavir if there was no test), they were younger than 18 years of age, had a history of severe kidney or liver disease, pregnancy, use of remdesivir, or, within 30 days before their test, were in hospital for any reason. Detailed exclusion criteria are provided in eAppendix 2 in Supplement 1.

Individuals prescribed nirmatrelvir and ritonavir were matched to individuals with COVID-19 who were not prescribed nirmatrelvir and ritonavir. Nirmatrelvir and ritonavir–exposed individuals were not required to have a positive PCR test, but all non–nirmatrelvir and ritonavir–exposed individuals required a positive test. Before matching, to minimize potential confounding, high-dimensional propensity score (HDPS) models were estimated for each of the 4 groups.^10^ The HDPS algorithm empirically selected covariates from the 730-day period prior to the COVID-19 test sample collection date. Within each cohort, nirmatrelvir and ritonavir–exposed individuals were matched 1-to-1 with non–nirmatrelvir and ritonavir–exposed individuals, without replacement, using the nearest neighbor method. Parameters used for matching were age (±2 years), sex, propensity score (±0.2 multiplied by the standard deviation of the pooled standard deviation of the logit of the propensity score),^11^ and year and calendar month (±1 month) of COVID-19–positive test.

Follow-up for study outcomes in an nirmatrelvir and ritonavir–exposed individual began on their nirmatrelvir and ritonavir initiation date. For each unexposed matched control participant, to avoid immortal time bias, follow-up began on the individual’s COVID-19 test collection date, plus the number of days that lapsed between the test collection date and treatment initiation in the control individual’s nirmatrelvir and ritonavir–exposed counterpart. Where the nirmatrelvir and ritonavir–exposed individual in a match did not have a PCR test, follow-up in the non–nirmatrelvir and ritonavir–exposed individual began 3 days after their PCR test sample collection date.

### Identification of Outcome Events

The primary outcome was a composite outcome of COVID-19–related emergency hospital visit or admission, or death from any cause, within 28 days of an individual’s cohort entry date. A secondary outcome was emergency department visit for any reason, with or without a subsequent admission to hospital. Estimation of hospitalization outcomes relied on emergency department (ED) records after March 31, 2022. This was because complete hospital discharge abstract data for the remainder of study period will not be available until late 2023. Instead, COVID-19–related emergency admissions after March 31, 2022, were identified using ED visit records from the NACRS database, which was complete for our study period. A NACRS record was counted as a COVID-19–related emergency hospitalization if it indicated COVID-19 infection (*International Statistical Classification of Diseases and Related Health Problems, Tenth Revision*, codes U07.1 and U07.2) and subsequent admission to hospital. To assess the reliability of this approach, we compared complete hospital discharge records with NACRS records in the year before our study. From March 1, 2021, to February 28, 2022, a NACRS record that indicated subsequent admission to hospital was 88.5% sensitive and 92.3% specific for the presence of an emergency hospital admission record in the hospital discharge abstract database.

### Sensitivity and Subgroup Analyses

Several sensitivity and subgroup analyses were performed. The primary outcome was further analyzed in subgroup analyses of COVID-19 vaccination history, age 70 years or older, sex, history of diabetes, and history of kidney disease. To avoid confounding by indication, we needed to ensure that all people entering the study were infected with COVID-19. The widespread and unrecorded use of rapid antigen tests during the study period required restricting non–nirmatrelvir and ritonavir–exposed control individuals to those who received positive PCR tests. Nirmatrelvir and ritonavir–exposed individuals did not require a positive PCR test because COVID-19 was the only indication for the drug, and prescribing physicians were required to make sure their patients tested positive within 5 days prior to prescribing nirmatrelvir and ritonavir. A sensitivity analysis was conducted in which nirmatrelvir and ritonavir–exposed individuals were also required to have a positive COVID-19 PCR test.

### Statistical Analysis

After assembling nirmatrelvir and ritonavir exposure and outcome data for the matched cohorts in 2 × 2 tables, we estimated the 28-day risk difference (RD) and the 28-day relative risk (RR) for each study outcome. These parsimonious analyses allowed for the straightforward reporting of RDs and numbers-needed-to-treat but did not account for the possible influence of competing risks. The reasonability of this approach was checked by comparing the RR estimates with hazard ratios estimated using the method of Fine and Gray^12^ and by estimating outcome-specific hazard functions.^13^ Both of those methods impose a proportional hazards assumption, which was checked using cumulative sums of Martingale-based residuals.^14^ Data analysis was conducted in SAS version 7.15 (SAS Institute). We used a 95% CI that excluded the null as our level of statistical significance.

## Results

There were 6866 individuals included in the study, of whom 3888 (56.6%) were female and whose median (IQR) age was 70 (57-80) years. Characteristics of the matched vulnerability groups are shown in Table 1. Nirmatrelvir and ritonavir–exposed individuals were balanced on sex and age in all 4 groups. Nirmatrelvir and ritonavir–exposed individuals were numerically less likely to have been vaccinated against COVID-19, a difference that was statistically significant in the EXEL group (difference −5.1%, 95% CI, −9.3% to −0.9%). Overall, vaccination was greater than 90% in the 3 CEV groups but less than 80% in the EXEL group. History of severe respiratory disorders, primary immunodeficiency (moderate or severe), transplant (solid organ or bone), immunosuppressive drug use, cancer, diabetes, nonsevere kidney conditions, heart failure, stroke, and neurological conditions were also compared for each matched group. Data on the first 3 could not be shown to protect privacy. For the remaining conditions, there were no statistically significant differences, with the exception of more nirmatrelvir and ritonavir–exposed individuals with neurological conditions in the CEV1 group (difference, 7.2%; 95% CI, 1.6% to 12.8%), more immunosuppressive drug use by nirmatrelvir and ritonavir–exposed individuals in the CEV2 group (difference, 1.6%; 95% CI, 0.3% to 2.9%), fewer nirmatrelvir and ritonavir–exposed individuals with nonsevere kidney conditions in the CEV3 group (difference, −6.5%; 95% CI, −10.7% to −2.4%), and fewer nirmatrelvir and ritonavir–exposed individuals who had a history of stroke (difference, −2.5%; 95% CI, −4.5% to −0.5%). Study flowcharts for each group are provided in eAppendix 3 in Supplement 1.

**Table 1.** Baseline Characteristics for the Matched Study Cohorts^a^

Characteristics	Individuals, No. (%)							
	CEV1		CEV2		CEV3		Expanded eligibility	
	Exposed (n = 280)	Unexposed (n = 280)	Exposed (n = 1314)	Unexposed (n = 1314)	Exposed (n = 1050)	Unexposed (n = 1050)	Exposed (n = 789)	Unexposed (n = 789)
Age, median (IQR), y	61 (46-74)	61 (46-74)	63 (49-76)	63 (49-76)	73 (59-83)	73 (59-83)	79 (72-86)	79 (72-86)
Sex								
Female	179 (63.9)	179 (63.9)	795 (60.5)	795 (60.5)	550 (52.4)	550 (52.4)	420 (53.2)	420 (53.2)
Male	101 (36.1)	101 (36.1)	519 (39.5)	519 (39.5)	500 (47.6)	500 (47.6)	369 (46.8)	369 (46.8)
Time since positive SARS-CoV-2 test^b^								
≤3 d	278 (99.3)	278 (99.3)	1295 (98.6)	1295 (98.6)	1038 (98.9)	1038 (98.9)	767 (97.2)	767 (97.2)
Prior SARS-CoV-2 vaccination								
No	7 (2.5)	NA^c^	72 (5.5)	59 (4.5)	75 (7.1)	58 (5.5)	210 (26.6)	170 (21.5)
Yes	273 (97.5)	275 (98.2)	1242 (94.5)	1255 (95.5)	975 (92.9)	992 (94.5)	579 (73.4)	619 (78.5)
No. of SARS-CoV-2 vaccine shots								
0	NA^c^	NA^c^	72 (5.5)	59 (4.5)	75 (7.1)	58 (5.5)	210 (26.6)	170 (21.5)
1	NA^c^	NA^c^	14 (1.1)	16 (1.2)	12 (1.1)	8 (0.8)	11 (1.4)	7 (0.9)
2	19 (6.8)	34 (12.1)	147 (11.2)	162 (12.3)	69 (6.6)	111 (10.6)	82 (10.4)	107 (13.6)
3	138 (49.3)	136 (48.6)	680 (51.8)	743 (56.5)	505 (48.1)	517 (49.2)	241 (30.5)	281 (35.6)
≥4	114 (40.7)	100 (35.7)	401 (30.5)	334 (25.4)	389 (37)	356 (33.9)	245 (31.1)	224 (28.4)
COVID-19 monoclonal antibody treatment								
No	280 (100)	280 (100)	1314 (100)	1314 (100)	1050 (100)	1050 (100)	789 (100)	789 (100)
Conditions^d^								
Immunosuppressive drug use	145 (51.8)	124 (44.3)	1288 (98)	1267 (96.4)	43 (4.1)	48 (4.6)	44 (5.6)	31 (3.9)
Cancer	41 (14.6)	37 (13.2)	154 (11.7)	139 (10.6)	113 (10.8)	97 (9.2)	9 (1.1)	NA^c^
Diabetes	61 (21.8)	61 (21.8)	263 (20)	251 (19.1)	434 (41.3)	412 (39.2)	298 (37.8)	273 (34.6)
Kidney condition (nonsevere)	77 (27.5)	84 (30)	345 (26.3)	373 (28.4)	367 (35)	436 (41.5)	360 (45.6)	357 (45.2)
Heart failure	19 (6.8)	18 (6.4)	106 (8.1)	86 (6.5)	100 (9.5)	106 (10.1)	181 (22.9)	149 (18.9)
Stroke	11 (3.9)	14 (5)	41 (3.1)	58 (4.4)	48 (4.6)	75 (7.1)	100 (12.7)	96 (12.2)
Neurological condition	47 (16.8)	27 (9.6)	49 (3.7)	41 (3.1)	43 (4.1)	48 (4.6)	68 (8.6)	84 (10.6)

Abbreviations: CEV, clinically extremely vulnerable; NA, not applicable.

^a^ Cohorts were matched on age (±2 years), sex, propensity score, and year and month of cohort entry (±1 month).

^b^ A value of 3 days was imputed for exposed individuals without a recorded positive SARS-CoV-2 PCR test within 5 days prior to nirmatrelvir and ritonavir dispensing.

^c^ Denotes a number less than 5.

^d^ Definitions for baseline conditions are provided in eAppendix 1 in Supplement 1.

Results for the association between nirmatrelvir and ritonavir and risk of death or COVID-19–related emergency hospitalization, the primary end point, are shown in Table 2. There was a statistically significant RD of −2.5% in the CEV1 group (95% CI, −4.8% to −0.2%), a −1.7% RD in the CEV2 group (95% CI, −2.9% to −0.5%), a nonstatistically significant −1.3% RD in the CEV3 group (95% CI, −2.8% to 0.1%), and a nonstatistically significant RD of 1.0% in the EXEL group (95% CI, −0.9% to 2.9%). Cumulative incidence functions are shown in the Figure for the CEV2, CEV3 and EXEL groups (to protect privacy, the CEV1 group could not be displayed). The Fine and Gray subdistribution hazard ratios and outcome-specific hazard ratios accounting for competing risks were the same or similar to the 28-day RR estimates from the fixed cohort analysis (eAppendix 4 in Supplement 1).

**Table 2.** Risk of Death or COVID-19–Related Emergency Hospitalization (Primary End Point) by Cohort

Group	Nirmatrelvir and ritonavir exposed, No.	Event, No.	Nirmatrelvir and ritonavir unexposed, No.	Event, No.	Risk difference, % (95% CI)	No. needed to treat	Relative risk (95% CI)
CEV1	280	NA^a^	280	NA^a^	−2.5 (−4.8 to −0.2)	40	0.22 (05 to 12)
CEV2	1314	23	1314	45	−1.7 (−2.9 to −0.5)	60	0.51 (0.31 to 0.84)
CEV3	1050	25	1050	39	−1.3 (−2.8 to 0.1)	75	0.64 (0.39 to 15)
EXEL	789	35	789	27	1 (−0.9 to 2.9)	99^b^	1.30 (0.79 to 2.12)

Abbreviations: CEV, clinically extremely vulnerable; EXEL, expanded eligibility.

^a^ Number masked to preserve privacy.

^b^ Number needed to treat is for harm, but not statistically significant.

**Figure.**
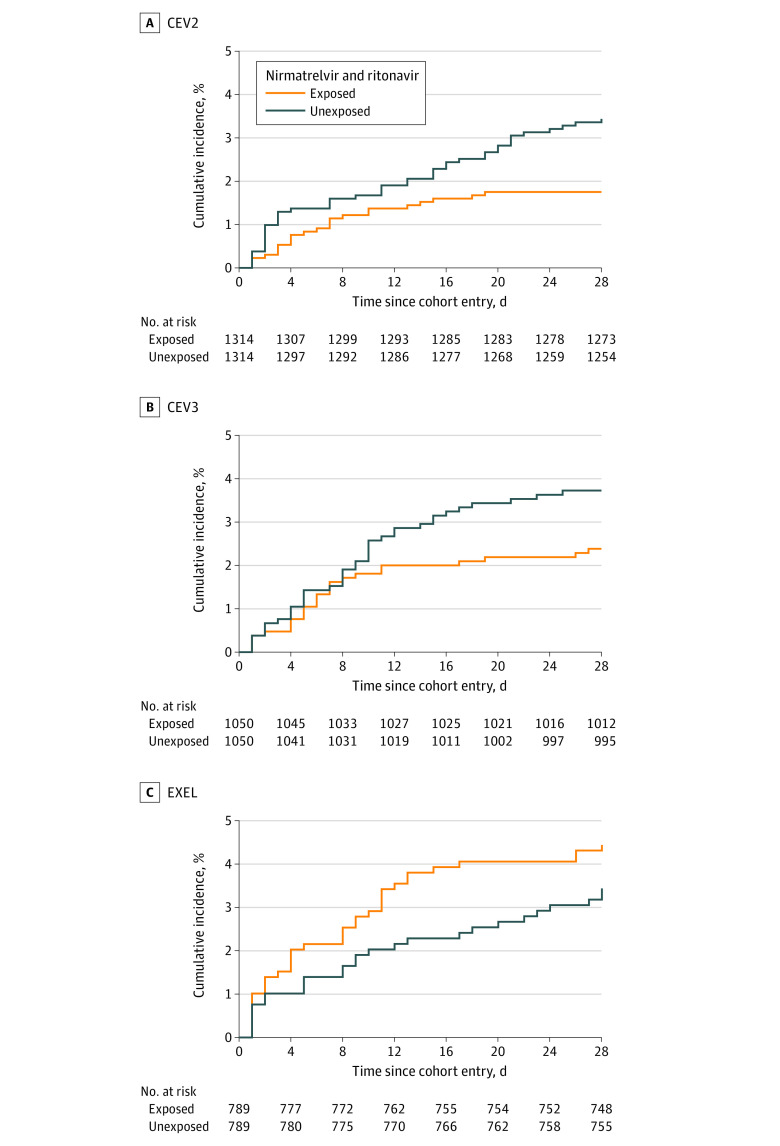
Cumulative Incidence of Death or COVID-19–Related Emergency Hospitalization CEV indicates clinically extremely vulnerable; EXEL, expanded eligibility.

In Table 3, the primary outcome is reported for the subgroups of individuals who were age 70 years or older and for male compared with female participants. RDs were nominally, but not statistically significantly, further from the null in the subgroup aged 70 years or older compared with the main analysis. RDs were nominally more protective in male compared with female participants, but these differences were also not statistically significant. Detailed data could not be presented for the CEV1 groups because of restrictions on reporting small numbers of events. Although event counts could not be reported, in individuals aged 70 years or older, the RD in 98 nirmatrelvir and ritonavir–exposed and 98 unexposed individuals was −8.2% (95% CI, −13.6% to −2.7%). In the CEV1 group, 179 nirmatrelvir and ritonavir–exposed women were matched to 179 unexposed women, and 101 nirmatrelvir and ritonavir–exposed men were matched to 101 unexposed men. Respectively, the RDs in these subgroups were −1.1% (95% CI, −2.7% to 0.4%), and −5.0% (95% CI, −10.6% to 0.7%). Among vaccinated individuals, RD estimates in the 4 vulnerability groups were similar to estimates for the whole study group. Small cell restrictions prevented reporting of the association in unvaccinated individuals in the 3 CEV groups. However, in the EXEL group, there were 7 events in 210 nirmatrelvir and ritonavir–exposed unvaccinated individuals and 6 events in 170 non–nirmatrelvir and ritonavir–exposed unvaccinated individuals. The RD in this subgroup was −0.2% (95% CI, −3.9% to 3.5%). There were no statistically significant differences between nirmatrelvir and ritonavir–exposed and unexposed individuals in ED visits (Table 4).

**Table 3.** Risk of Death or COVID-19–Related Emergency Hospitalization, by Sex and in Individuals Aged 70 Years or Older

Group^a^	Nirmatrelvir and ritonavir exposed, No.	Event, No.	Nirmatrelvir and ritonavir unexposed, No.	Event, No.	Risk difference, % (95% CI)	No. needed to treat
Among individuals ≥70 y						
CEV2	510	20	498	35	−3.1 (−5.9 to −0.3)	33
Among female and male subgroups (all ages)						
CEV2, female	795	11	795	20	−1.1 (−2.5 to 0.2)	89
CEV2, male	519	12	519	25	−2.5 (−4.8 to −0.3)	40
CEV3, female	550	15	550	17	−0.4 (−2.3 to 1.6)	275
CEV3, male	500	10	500	22	−2.4 (−4.6 to −0.2)	42
EXEL, female	420	17	420	10	1.7 (−0.7 to 4)	60^b^
EXEL, male	369	18	369	17	0.3 (−2.8 to 3.3)	369^b^
Among individuals with diabetes						
CEV2	263	10	251	9	0.2 (−3 to 3.5)	462^b^
CEV3	434	13	412	21	−2.1 (−4.8 to 0.6)	48
EXEL	298	13	273	11	0.3 (−3 to 3.6)	301^b^
Among individuals with kidney disease						
CEV2	345	8	373	26	−4.7 (−7.7 to −1.6)	22
CEV3	367	12	436	27	−2.9 (−5.8 to 0)	35
EXEL	360	17	357	17	0 (−3.2 to 3.1)	NA
Among individuals vaccinated against COVID-19						
CEV2	1242	20	1255	43	−1.8 (−3 to −0.6)	56
CEV3	975	24	992	37	−1.3 (−2.8 to 0.3)	79
EXEL	579	27	619	21	1.3 (−1 to 3.5)	79^b^

Abbreviations: CEV, clinically extremely vulnerable; EXEL, expanded eligibility; NA, not applicable.

^a^ Events for the CEV1 groups cannot be reported because of small cell restrictions. Among individuals aged 70 years or older, the risk difference was −8.2% (95% CI −13.4% to −2.7%).

^b^ Number needed to treat is for harm, but not statistically significant.

**Table 4.** Risk of Any Emergency Department Visit, by Group

Group	Nirmatrelvir and ritonavir exposed, No.	Event, No.	Nirmatrelvir and ritonavir unexposed, No.	Event, No.	Risk difference, % (95% CI)	No. needed to treat	Relative risk (95% CI)
CEV1	264	22	264	29	−2.7 (−7.7 to 2.4)	38	0.76 (0.45 to 1.29)
CEV2	1259	93	1259	115	−1.7 (−3.9 to 0.4)	58	0.81 (0.62 to 15)
CEV3	1018	64	1018	62	0.2 (−1.9 to 2.3)	510^a^	13 (0.74 to 1.45)
EXEL	739	53	739	46	0.9 (−1.6 to 3.5)	106^a^	1.15 (0.79 to 1.69)

Abbreviations: CEV, clinically extremely vulnerable; EXEL, expanded eligibility.

^a^ Number needed to treat is for harm, but not statistically significant.

In the main analysis, nirmatrelvir and ritonavir–exposed individuals did not require a PCR test because COVID-19 was the only indication for nirmatrelvir and ritonavir. A sensitivity analysis that required all nirmatrelvir and ritonavir–exposed individuals to also have a positive PCR test resulted in a subgroup of 1362 individuals from the original 6866 study population. A small number of events prevented reporting the sensitivity analysis in the CEV1 group. In 480 individuals in the CEV2 group, the estimated RD was the same as in the main analysis at −1.7% (95% CI, −5.6% to 2.2%). In 352 individuals in the CEV2 group, the RD was −1.1% (95% CI, −6.0% to 3.7%), compared with −1.3% in the main analysis. In 470 individuals in the EXEL group, the RD was 3.4% (95% CI, −1.3% to 8.1%), compared with 1.0% in the main analysis.

## Discussion

In this observational study of 6866 individuals in BC, nirmatrelvir and ritonavir exposure was associated with a lower risk of death or COVID-19–related hospitalization in individuals with extreme vulnerability to complications from COVID-19. The same association was not observed in lower-risk individuals in the EXEL group, a result that was robust across sex and older vs younger age. The results from our CEV cohorts were compatible with the results of the EPIC-HR trial,^2^ where a statistically significant reduction of hospitalization or death was seen. The results in the lower-risk EXEL group appeared comparable with the unpublished results of the EPIC-SR trial,^4^ where no difference in the primary outcome was seen, although we observed a nominally higher hospitalization and mortality rate in those exposed to nirmatrelvir and ritonavir. With one important exception, our results were generally consistent with observational studies of nirmatrelvir and ritonavir,^5,6^ where the benefit of nirmatrelvir and ritonavir is driven by high-risk groups within an exposed population. The important exception in our study was that, owing to the stratified approach in our analysis, older age (≥70 years) did not have a statistically significant beneficial association with nirmatrelvir and ritonavir after accounting for other comorbidities.

### Strengths and Limitations

Our study has several important strengths. First, nirmatrelvir and ritonavir was assessed in stratified analyses in cohorts that were based on different degrees of vulnerability to COVID-19–related morbidity and in secondary analyses of age and sex. This approach prevented overly parsimonious inferences in the presence of effect estimate modification by these factors. If all vulnerability groups had been combined into one cohort, the RD for the primary outcome in the subgroup of individuals aged 70 years or older would have been −1.4% and statistically significant. However, our analysis showed that individuals aged 70 years or older of age in the EXEL group did not have their risk of the outcome reduced by nirmatrelvir and ritonavir, which is an important finding that could prevent overtreatment with nirmatrelvir and ritonavir in older people who are not extremely vulnerable to complications from COVID-19. Second, use of reference groups with similar COVID-19 vulnerability and who tested positive for COVID-19 close to the time of their matched nirmatrelvir and ritonavir–exposed counterpart avoided confounding by indication and by COVID-19 variant. Third, use of HDPS in the matching algorithms allowed for control of empirical residual confounding by a constellation of factors that could not have been achieved by means of statistical adjustment, or by weighting, because events were sufficiently rare to have limited the number of confounding variables that could have been accommodated with those analytical approaches.

Several limitations of our analyses merit mention. Omicron was the main circulating variant of COVID-19 during the study period, and our results may therefore not be applicable to other variants, past or future. Omicron is associated with less severe disease than the Delta variant, which was prevalent during the EPIC-HR trial era. This could be one reason the estimates in our study were attenuated by comparison. Our analyses relied on administrative claims data that may not have captured all factors used by physicians in choosing treatment with nirmatrelvir and ritonavir. To limit the influence from this possible source of bias, we excluded nirmatrelvir and ritonavir–exposed individuals and other individuals with COVID-19 who did not have data indicating which of the 4 vulnerability groups they belonged to. We then analyzed study outcomes stratified by these groups. Future research might focus on creating an approach to assessing COVID-19 vulnerability that is more granular than the 4 vulnerability groups used in this study. Such an approach has the prospect of offering more targeted treatment with nirmatrelvir and ritonavir. More individuals in the nirmatrelvir and ritonavir–exposed group of the EXEL group were unvaccinated (26.6% vs 21.5%). The RD for the primary outcome in the EXEL group was 1.0%, which equates to an RR of 1.30. Even if the independent RR of the primary outcome in nonvaccinated vs vaccinated non–nirmatrelvir and ritonavir–exposed individuals in the EXEL group was an un unrealistic RR of 100, the RR for the primary outcome in the EXEL group in nirmatrelvir and ritonavir–exposed vs unexposed individuals would have been at most 1.23. Thus, the imbalance in vaccination cannot account for the reported association. Our cohort criteria required a positive COVID-19 PCR test. This may have limited the generalizability of our findings.

## Conclusions

In this cohort study of individuals with COVID-19 in BC between February 1, 2022, and February 3, 2023, use of nirmatrelvir and ritonavir appeared to reduce the risk of the composite outcome of death or COVID-19–related hospitalization in high-risk, mostly moderately or severely immunocompromised individuals. Stratified analysis of individuals according to vulnerability to complications from COVID-19 is crucial to understanding which individuals should use nirmatrelvir and ritonavir. In our study, lower-risk individuals, including those older than 70 years who were not moderately or severely immunocompromised, did not appear to benefit from nirmatrelvir and ritonavir.

## Notes

Supplement 1. **eAppendix 1.** Clinical Vulnerability Definitions and Lookback Periods **eAppendix 2.** Study Exclusion Criteria **eAppendix 3.** Study Flowcharts **eAppendix 4.** Fine and Gray and Outcome-Specific Hazard Ratios

Supplement 2.
Data Sharing Statement
