# Errors in Tables

**DOI:** 10.1001/jamanetworkopen.2024.1976

**Published:** 2024-02-14

In the Original Investigation titled “Nirmatrelvir-Ritonavir and COVID-19 Mortality and Hospitalization Among Patients With Vulnerability to COVID-19 Complications,”^1^ published October 2, 2023, there were errors in Tables 2 and 4. In Table 2, the relative risk for the clinically extremely vulnerable group 1 (CEV1) should have read 0.22 (95% CI, 0.05-1.02), and the relative risk for CEV3 should have read 0.64 (95% CI, 0.39-1.05). In Table 4, the relative risk for CEV2 should have read 0.81 (95% CI, 0.62-1.05), and the relative risk for CEV3 should have read 1.03 (95% CI, 0.74-1.45). This article has been corrected.^1^
