# Supplementary material for: Nirmatrelvir-Ritonavir and COVID-19 Mortality and Hospitalization Among Patients With Vulnerability to COVID-19 Complications
Source: JAMA Netw Open. 2023 Oct 2;6(10):e2336678. doi: 10.1001/jamanetworkopen.2023.36678 (PMC10546233; doi:10.1001/jamanetworkopen.2023.36678)
Supplement: Supplement 1. — eAppendix 1. Clinical Vulnerability Definitions and Lookback Periods eAppendix 2. Study Exclusion Criteria eAppendix 3. Study Flowcharts eAppendix 4. Fine and Gray and Outcome-Specific Hazard Ratios [file jamanetwopen-e2336678-s001.pdf]

Supplemental Online Content

Dormuth CR, Kim JD, Fisher A, Piszczek J, Kuo IF. Nirmatrelvir-ritonavir and COVID-19 mortality and hospitalization among patients with vulnerability to COVID-19 complications. *JAMA Netw Open.* 2023;6(10):e2336678. doi:10.1001/jamanetworkopen.2023.36678

eAppendix 1. Clinical Vulnerability Definitions and Lookback Periods

eAppendix 2. Study Exclusion Criteria

eAppendix 3. Study Flowcharts

eAppendix 4. Fine and Gray and Outcome-Specific Hazard Ratios

This supplemental material has been provided by the authors to give readers additional information about their work.

© 2023 Dormuth CR et al. *JAMA Network Open.*

eAppendix 1. Clinical Vulnerability Definitions and Lookback Periods

Definitions for the CEV1, CEV2, CEV3 and EXEL groups are provided below. Publicly available information on these groups is also available for the CEV groups at http://www.bccdc.ca/Health-Professionals-Site/Documents/COVID-treatment/ClinicalPracticeGuide_Therapeutics_MildModerateCOVID.pdf, and for the EXEL group at http://www.bccdc.ca/Health-Professionals-Site/Documents/COVID-treatment/PracticeTool2_CEVCriteria.pdf.

CEV 1: Severely immunosuppressed individuals

The first CEV group contains individuals 18 years of age and older severely immunosuppressed due to one or more of the conditions listed below. Among individuals age 18 years or older at cohort entry, the following conditions for inclusion in CEV group 1 were used:

1) Solid organ transplant
    a. Presence of a diagnosis, procedure or fee code for solid organ transplant of kidney, liver, lung, heart, pancreas or islet cell, bowel or combination at any time before (or on) cohort entry date.
2) Treated for malignant hematologic conditions
    a. Presence of a diagnosis code for a hematologic condition AND a procedure or fee code for chemotherapy or immunotherapy in the last year before cohort entry OR
    b. Presence of a primary diagnosis code for a hematologic condition in a hospital episode in the last year before cohort entry.
3) Bone marrow, stem cell transplant or transplant-related immunosuppressant use
    a. Presence of a diagnosis, procedure or fee code for bone marrow transplant or stem cell transplant in the last two years before cohort entry OR
    b. Dispensing or remaining days supply of an immunosuppressant in the last three months AND
        i. Presence of a diagnosis, procedure or fee code for bone marrow transplant or stem cell transplant in the last five years before cohort entry.
4) Anti-CD20 agents or B-cell depleting agents
    a. Dispensing of an anti-CD20 agent or B-cell depleting agent in the last two years before cohort entry.
5) Severe primary immunodeficiencies.
    a. Presence of a diagnosis code for a severe primary immunodeficiency in the last five years before cohort entry.
        i. Severe immunodeficiencies include combined immunodeficiencies affecting T-cells, immune dysregulation (particularly familial hemophagocytic lymphohistiocytosis) or those with type 1 interferon

© 2023 Dormuth CR et al. *JAMA Network Open.*

defects (caused by a genetic primary immunodeficiency disorder or secondary to anti-interferon autoantibodies).

ii. Note: there were no specific diagnosis codes for type 1 interferon defects; however, these may be included under "other immunodeficiencies" (ICD-10-CA: D84.x, D89.x; ICD-9-CM: 279.4. 279.9).

CEV 2: Moderately immunosuppressed individuals

The second CEV group contained individuals 18 years of age and older who were moderately immunosuppressed due to one or more of the conditions listed below. Among individuals aged 18 years or older at cohort entry date who were not identified in CEV group 1, the following conditions for inclusion in CEV group 2 were used.

1) Treatment for cancer including solid tumors
    a. Presence of a diagnosis, procedure or fee code for chemotherapy or immunotherapy (i.e., systemic therapy) in the last six months OR
    ~~b.~~ Presence of a procedure or fee code for radiation therapy in the last three months before cohort entry.
2) Significantly immunosuppressing drugs
    a. Dispensing or remaining days supply of a biologic in the last three months OR
    b. Dispensing or remaining days supply of an oral immunosuppressing drug in the last month OR
    c. Dispensing or remaining days supply of an oral steroid (20mg/day of prednisone equivalent taken on an ongoing basis) in the last month OR
    d. Dispensing of an immune-suppressing infusion or injection in the last three months before cohort entry.
3) Advanced untreated HIV infection or treated HIV
    a. Presence of a diagnosis code (2 MSP or 1 DAD/NACRS) for AIDS at any time before cohort entry OR
    b. Presence of 1 MSP diagnosis for AIDS within 2 weeks after a CD4 lab test OR
    c. Presence of a CD4 lab test result with CD4 count ≤ 200/mm^3 OR CD4 fraction ≤ 15% at any time before cohort entry.
        i. Use conversion factor of 10^3 to convert CD4 count units in PLIS data from 10^9 per L to per mm^3.
4) Moderate primary immunodeficiencies
    a. Presence of diagnosis code for a primary immunodeficiency with a genetic cause at any time before cohort entry OR
    b. Presence of diagnosis code for a primary immunodeficiency AND presence of a procedure code for immunoglobulin replacement therapy in the last year before cohort entry.

© 2023 Dormuth CR et al. *JAMA Network Open.*

5) Renal conditions
    a. Presence of a diagnosis, procedure or fee code for dialysis (hemodialysis or peritoneal dialysis) in the last 6 months before cohort entry OR
    b. Presence of a diagnosis code for severe kidney/renal disease in the last two years before cohort entry AND presence of an estimated glomerular filtration rate (eGFR) lab result < 15ml/min in the last two years before cohort entry OR
    c. Presence of a diagnosis code for glomerulonephritis AND dispensing of a steroid in the last two years before cohort entry.

CEV 3: Individuals with high-risk conditions

The third CEV group included individuals 18 years of age or older who were not immunosuppressed but who are at high risk of complications from SARS-COV-2 due to having one or more of the diseases listed below. The following conditions for inclusion in CEV group 3 were used for individuals not in the CEV1 or CEV2 groups:

1) Severe respiratory disorders
    a. Presence of a diagnosis code for cystic fibrosis at any time before cohort entry OR
    b. Dispensing of a Cystic Fibrosis Transmembrane Conductance Regulator (CFTR) modulator (Kalydeco, Orkambi, Symdeko, Trikafta)[1] at any time before cohort entry OR
    c. Dispensing of a specialized medication for pulmonary arterial hypertension or severe pulmonary fibrosis/interstitial lung disease in the last two years OR
    d. Presence of a primary diagnosis code for COPD in a hospital episode in the last year before cohort entry OR
    e. Presence of a primary diagnosis code for asthma in a hospital episode in the last year before cohort entry OR
    f. Dispensing of a biologic for asthma in the last three months and at least one of (within six months of dispensing):
        i. Procedure or fee code for therapeutic intervention on the respiratory system or ventilation
        ii. Diagnosis code for severe pulmonary arterial hypertension
        iii. Diagnosis code for severe pulmonary fibrosis/interstitial lung disease
2) Rare blood disorders
    a. Presence of a diagnosis code for sickle cell disease or hemolytic uremic syndrome in the last five years before cohort entry
3) Rare metabolic disorders

[1] Cystic Fibrosis Canada. Access to Medicines. Cystic Fibrosis Canada. Accessed 2022 Jul 05. Available from: https://www.cysticfibrosis.ca/our-programs/advocacy/access-to-medicines.

© 2023 Dormuth CR et al. *JAMA Network Open.*

    a. Presence of a diagnosis code for maple syrup urine disease, methylmalonic aciduria, urea cycle defect, or glutaric aciduria in the last five years before cohort entry

4) Splenectomy (anatomical or functional asplenia)
    a. Presence of a diagnosis code for asplenia or procedure or fee code for splenectomy at any time before cohort entry.
5) Diabetes treated with insulin
    a. Dispensing of insulin in the last two years before cohort entry
6) Hematological and other cancers not captured in CEV groups 1 or 2 (i.e., not on treatment but undergoing surveillance)
    a. Presence of a primary diagnosis code for cancer during a hospital episode in the last year OR visits to an oncologist in the last year before cohort entry.
7) Significant developmental disabilities
    a. Presence of a diagnosis code for Down's syndrome, cerebral palsy, or intellectual developmental disability at any time before cohort entry
8) Pregnant with a serious heart disease
    a. Presence of a diagnosis code for pregnancy or fee code for a prenatal visit in the last 3 months and at least one of:
        i. Diagnosis code for an acquired or congenital heart disease in the last two years before cohort entry OR
        ii. Two or more visits to a cardiologist within 9 months of the diagnosis or fee code from step a.
9) Neurological or other conditions causing significant muscle weakness around lungs
    a. Presence of a diagnosis code for a neurological condition AND
        i. Presence of a procedure or fee code for positive pressure ventilation in the last two years before cohort entry OR
        ii. Presence of a procedure code for other ventilation, intubation or respiration during a hospital episode in the last two years before cohort entry

EXPELIG: Expanded Eligibility Cohort

Eligibility for Paxlovid was expanded to include individuals 18 years of age or older who met certain definitions of age, comorbidity, vaccination status, and previous SARS-COV-2 infection. Using the following algorithm, we constructed a cohort of such individuals who were not identified in CEV groups 1 to 3:

1. Determine the number of SARS-COV-2 vaccine doses at any time before cohort entry date

© 2023 Dormuth CR et al. *JAMA Network Open.*

- Determine the maximum number of vaccine doses before cohort entry from the Provincial Immunization Registry.

2. Determine SARS-COV-2 infection at any time before cohort entry date (yes/no)
   - Presence of a positive SARS-COV-2 RNA lab test result.
   - Presence of a diagnosis code for SARS-COV-2 (ICD-10-CA: U071, U072, U073, U074, U075) in a hospital episode or emergency department visit.[2,3]
     - For emergency department visits, if both ICD-10-CA diagnosis codes U072 and Z038 are present for the same visit, then define as no SARS-COV-2 infection for that visit.[4]
3. Determine the number of chronic conditions/comorbidities
   - Obesity: Previous diagnosis of obesity in the last 2 years before cohort entry.
   - Smoking status: Previous physician or nurse practitioner visit with fee item related to education for smoking, diagnosis code related to tobacco use, or dispensing of a smoking cessation product in the last 2 years before cohort entry.
   - Diabetes: Previous diagnosis of type 1 or 2 diabetes mellitus, Hb A1c lab result with a value ≥ 6.5%, or the dispensing of insulin or an oral antidiabetic mediation in the last 2 years before cohort entry.
   - Heart failure: Previous diagnosis of heart failure in the last 2 years before cohort entry.
   - Heart disease: Previous diagnosis of acquired or congenital heart disease in the last 2 years before cohort entry.
   - Stroke: Previous diagnosis of stroke in the last 2 years before cohort entry.
   - Neurological conditions: Previous diagnosis of a neurological condition in the last 2 years before cohort entry.

After completing steps 1–3, assign individuals to the following subgroups:

- Unvaccinated and without previous infection and either: ≥ 50 years at cohort entry OR ≥ 3 chronic conditions/comorbidities
- ≥ 50 years at cohort entry with 1-2 vaccine doses or previous infection alone, AND ≥ 3 chronic conditions/comorbidities.
- ≥ 70 years at cohort entry with 1-2 vaccine doses or previous infection alone, AND ≥ 1 chronic conditions/comorbidities.

---

[2] CIHI. ICD-10-CA Coding Direction for SARS-COV-2 – Part 1: Questions and Answers. Canadian Institute for Health Information. Accessed 2022 Jun 29. Available from: https://www.cihi.ca/en/icd-10-ca-coding-direction-for-SARS-CoV-2-part-1-questions-and-answers#documentation.

[3] WHO. Emergency ICD codes for SARS-COV-2 disease outbreak. World Health Organization. Accessed 2022 Jun 29. Available from: https://www.who.int/classifications/classification-of-diseases/emergency-use-icd-codes-for-SARS-CoV-2-disease-outbreak.

[4] CIHI. SARS-COV-2: Locating the ICD-10-CA/CCI Code. Canadian Institute for Health Information. Accessed 2022 Jun 29. Available from: https://www.cihi.ca/sites/default/files/document/SARS-CoV-2-locating-icd-10-ca-cci-code-jobaid-en.pdf.

© 2023 Dormuth CR et al. *JAMA Network Open.*

- ≥ 70 years at cohort entry with ≥ 3 chronic conditions/comorbidities, regardless of vaccine status or previous infection
- Indigenous individuals (identified by enrollment in the First Nations MSP premium group in the last 5 years before cohort entry) who meet at least one of the following: unvaccinated without previous infection, ≥ 50 years with 1-2 vaccine doses or previous infection alone, OR ≥ 70 years regardless of vaccine status or previous infection.

© 2023 Dormuth CR et al. *JAMA Network Open.*

eAppendix 2. Study Exclusion Criteria

i. Individuals were not allowed to enter the cohort more than once. When individuals who at different times qualified as NMV-r-exposed and unexposed, the NMV-r instance was chosen.

ii. Prior to their cohort-qualifying COVID test or NMV-r prescription, individuals required at least 730 days of reasonably continuous enrollment in the BC Medical Services Plan.

iii. Less than 18 years of age at cohort entry (CEDT), or missing age

Note: The following exclusion criteria were applied after matching:

iv. Prior use of remdesivir

v. Hospital admission within 30 days prior to the CEDT

vi. Hospital separation within 30 days prior to the CEDT

vii. A diagnosis code for pregnancy in the 730 days prior to the CEDT.

ICD-9 Diagnostic Code list for MSP records

ICD-9	Description
630.x	Molar pregnancy
631.x	Other abnormal product of conception
632.x	Abortion, missed
633.x	Abdominal/fallopian/ovarian/ectopic pregnancy
634.x	Spontaneous abortion
635.x	Legally induced abortion
636.x	Illegally induced abortion
637.x	Unspecified abortion
V27.x	Single/Multiple liveborn
656.4	Intrauterine death affecting management of mother

ICD-10-CA Equivalent Codes

ICD-10-CA	Description
O00.x	Ectopic pregnancy
O01.x	Molar pregnancy
O02.x	Other abnormal products of conception
O03.x	Spontaneous abortion
O04.x	Complications following (induced) termination of pregnancy
O05.x	Other abortion
Z37.x	Outcome of delivery
O36.4x	Maternal care for intrauterine death

© 2023 Dormuth CR et al. *JAMA Network Open.*

eAppendix 3. Study Flowcharts

The source population was all 4,520,101 individuals who were residents of BC during the study period. There were 70,869 individuals (1.6%) in the source population who had a positive COVID-19 PCR test, or who received NMVr, or both. Of these, 17,971 (25.4%) were given NMVr, and 52,898 (74.6%) had a positive test but were not prescribed NMVr. To avoid confounding by indication, our study design required NMVr-exposed individuals to have a positive COVID-19 PCR test within 5 days prior to entering the study. Data were not available for rapid antigen tests, and 15,675 of 17,971 NMVr individuals (87.2%) had no record of being PCR tested within 5 days prior to receiving the treatment. Since COVID-19 was the only approved indication for NMVr during the study period, NMVr-exposed individuals in the study without a COVID-19 PCR test record (80.2% of 3,433 NMVr-exposed individuals) were included in the analysis and assumed to have been COVID-19 positive, with an imputed test sample collection date 3 days prior to being given NMVr.

From the 70,869 individuals who had a positive COVID-19 PCR test or who received NMVr (30,180 of 70,869; 42.6%), there were 3,794 (5.4%) who were identified as part of the CEV1 group, 13,290 (18.8%) who were part of the CEV2 group, 10,112 (14.3%) who belonged to the CEV3 group, and 8,960 (12.6%) who qualified for the EXEL group. There were 34,727 individuals (49.0%) who did not meet the criteria for any of the four vulnerability groups, according to the databases we used, and were excluded from further analysis. Most of these (28,951; 83.4%) were originally extracted because they had a positive COVID-19 PCR test during the study period and may not have met local eligibility criteria for NMVr. However, 5,776 (16.6%) were prescribed NMVr without evidence in the database of belonging to one of the vulnerability groups. After applying exclusion criteria and performing 1:1 matching of NMVr-exposed to unexposed individuals, there were 560 individuals in the CEV1 study group, 2,628 in the CEV2 group, 2,100 in the CEV3 group, and 1,578 in the EXEL group. Lists of diagnostic codes, procedure codes, WHO Anatomical Therapeutic Codes, laboratory test codes, and propensity score variables can be made available upon request.

© 2023 Dormuth CR et al. *JAMA Network Open.*

Figure S1. Patient flow chart for the CEV1 group

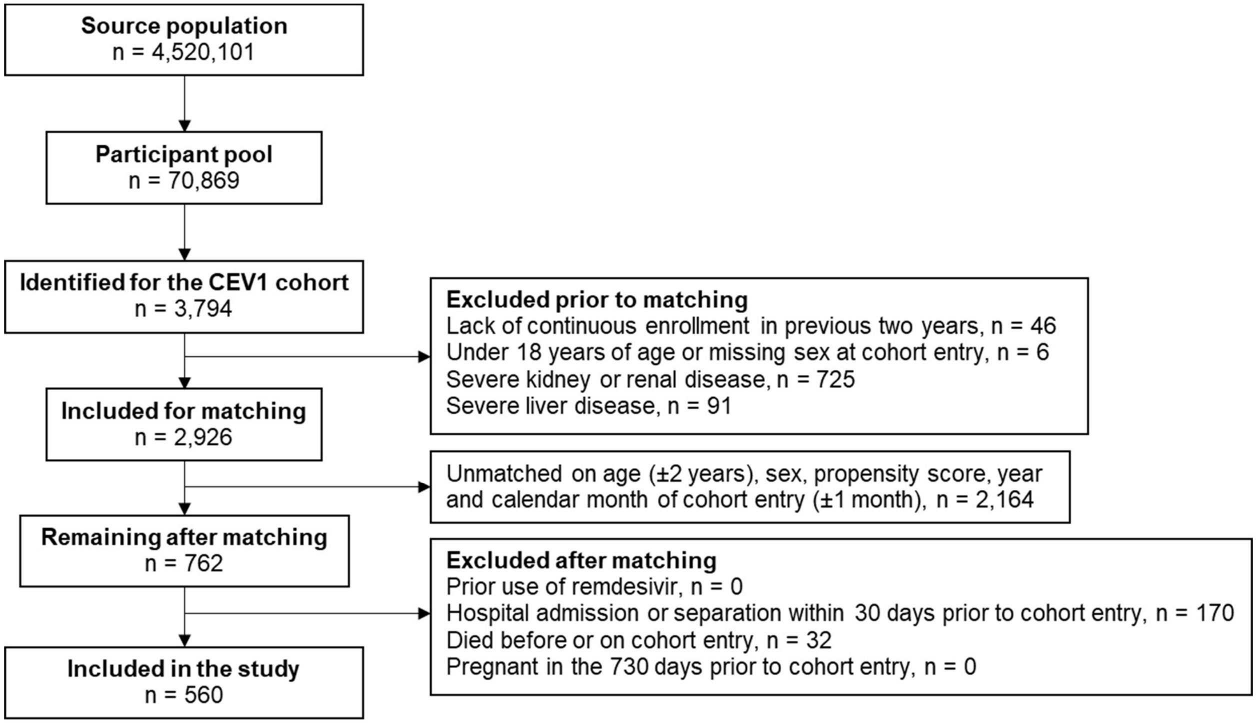

© 2023 Dormuth CR et al. *JAMA Network Open.*

Figure S2. Patient flow chart for the CEV2 group

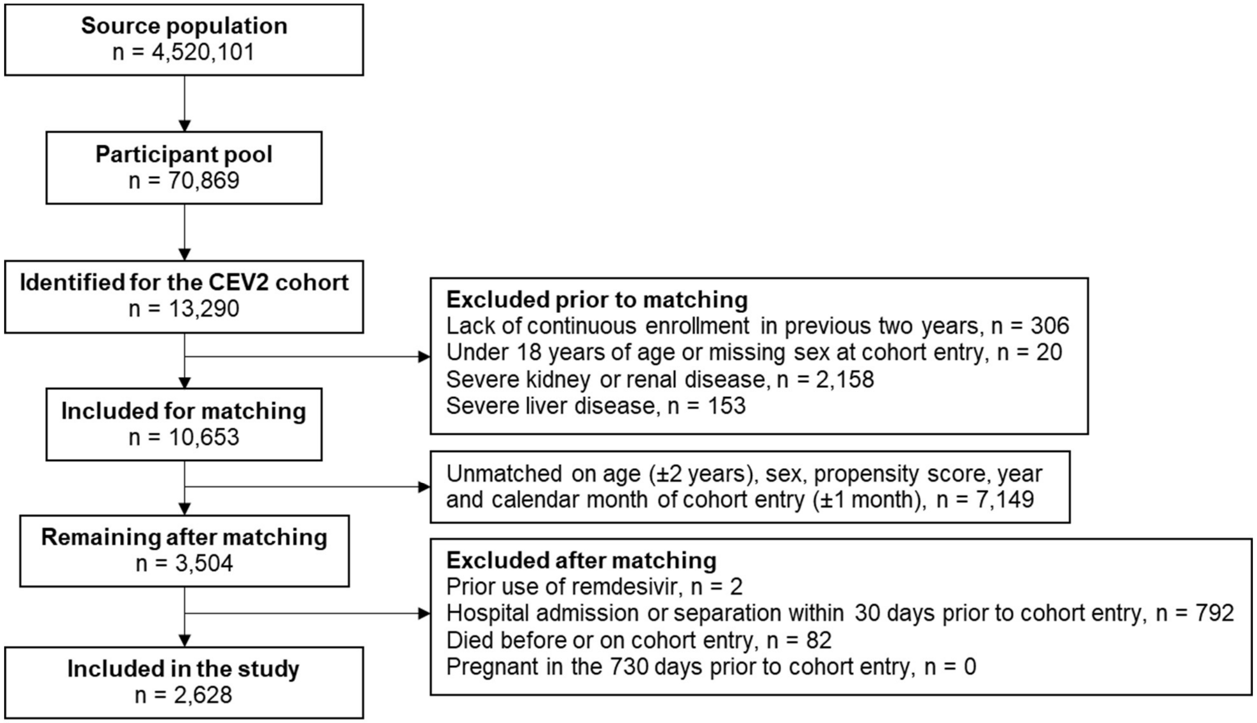

© 2023 Dormuth CR et al. *JAMA Network Open.*

Figure S3. Patient flow chart for the CEV3 group

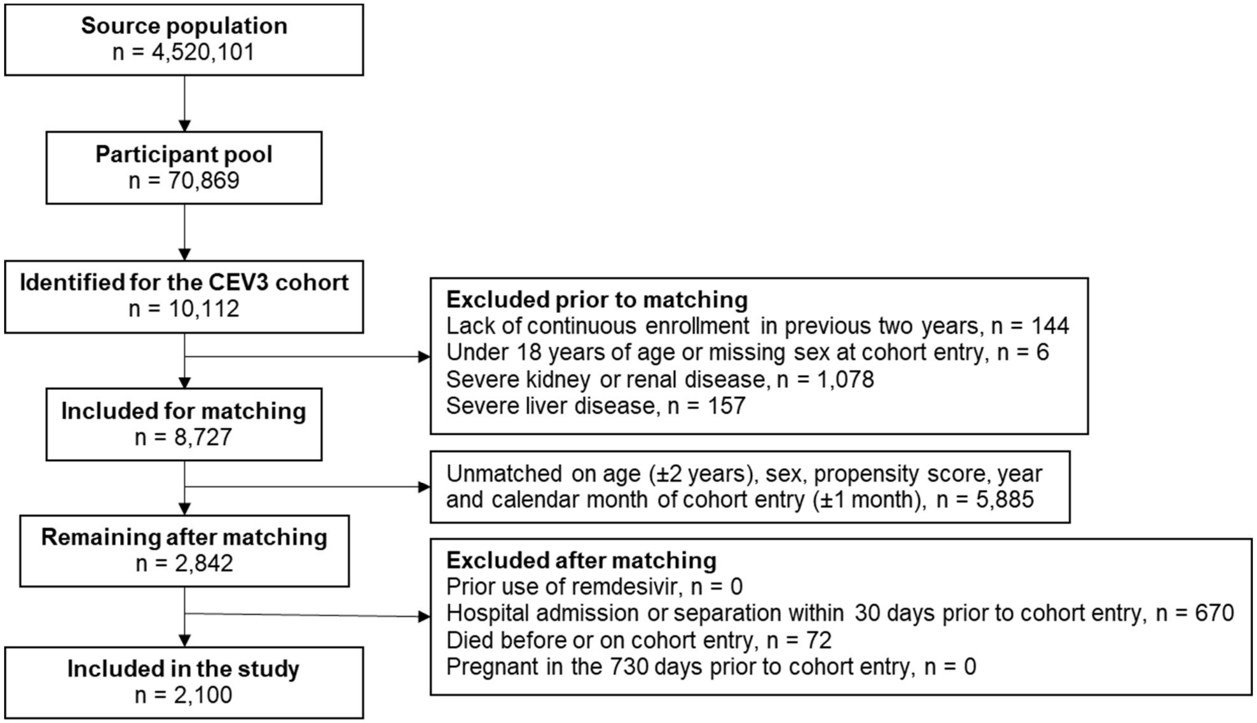

© 2023 Dormuth CR et al. *JAMA Network Open.*

Figure S4. Patient flow chart for the CEV4 group

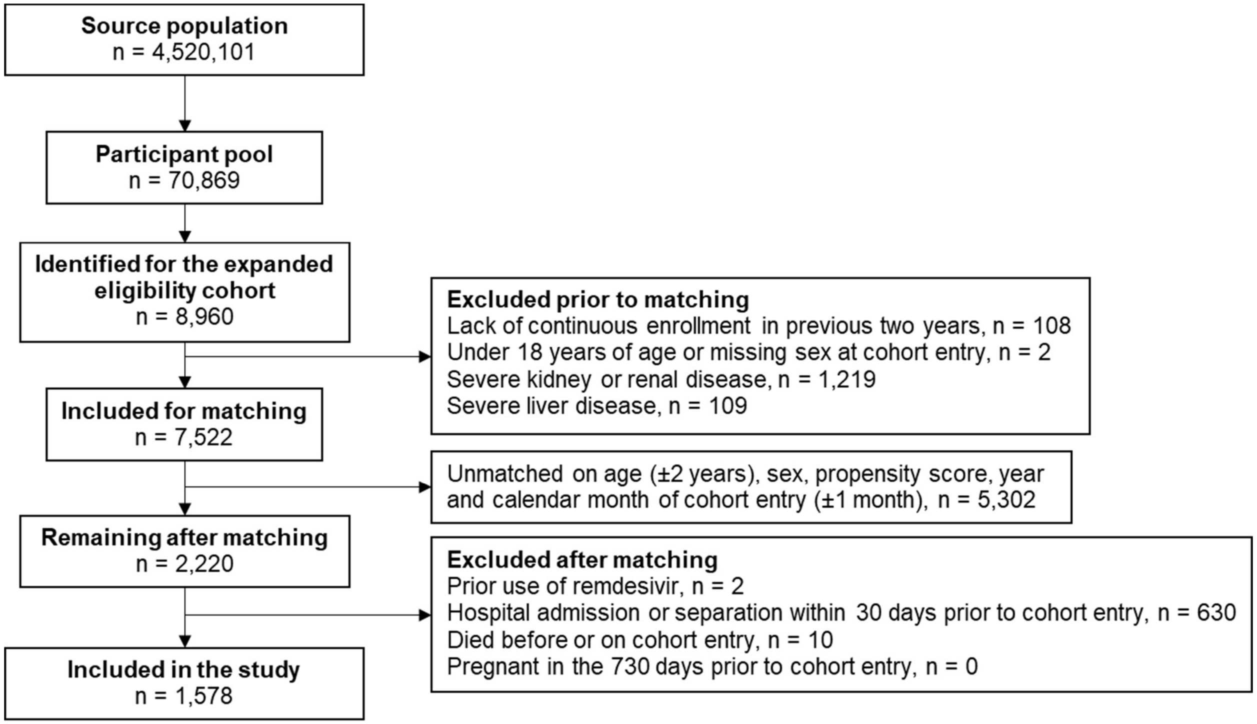

© 2023 Dormuth CR et al. *JAMA Network Open.*

eAppendix 4. Fine and Gray and Outcome-Specific Hazard Ratios

Supplement to Table 2. Fine & Gray hazard ratios and outcome-specific hazard ratios

Group	NMVr exposed	Event	NMVr unexposed	Event	Risk Difference % (95% CI)	Number Needed to Treat	Relative Risk (95% CI)	Hazard Ratio (Fine & Gray)	Hazard Ratio (outcome-specific)
CEV1	280	s	280	s	-2.5 (-4.8, -0.2)	40	0.22 (0.05, 1.02)	0.22	0.22
CEV2	1314	23	1314	45	-1.7 (-2.9, -0.5)	60	0.51 (0.31, 0.84)	0.51	0.51
CEV3	1050	25	1050	39	-1.3 (-2.8, 0.1)	75	0.64 (0.39, 1.05)	0.64	0.64
EXEL	789	35	789	27	1.0 (-0.9, 2.9)	99*	1.30 (0.79, 2.12)	1.31	1.30

* Number needed to treat is for harm, but not statistically significant. 's' denotes a number that was masked to preserve privacy.

Supplement to Table 4. Fine & Gray hazard ratios and outcome-specific hazard ratios

Group	NMVr exposed	Event	NMVr unexposed	Event	Risk Difference % (95% CI)	Number Needed to Treat	Relative Risk (95% CI)	Hazard Ratio (Fine & Gray)	Hazard Ratio (cause-specific)
CEV1	264	22	264	29	-2.7 (-7.7, 2.4)	38	0.76 (0.45, 1.29)	0.75	0.74
CEV2	1259	93	1259	115	-1.7 (-3.9, 0.4)	58	0.81 (0.62, 1.05)	0.80	0.80
CEV3	1018	64	1018	62	0.2 (-1.9, 2.3)	510*	1.03 (0.74, 1.45)	1.03	1.02
EXEL	739	53	739	46	0.9 (-1.6, 3.5)	106*	1.15 (0.79, 1.69)	1.16	1.16

* Number needed to treat is for harm, but not statistically significant.

© 2023 Dormuth CR et al. *JAMA Network Open.*
