# Supplementary material for: Nirmatrelvir-Ritonavir and COVID-19 Mortality and Hospitalization Among Patients With Vulnerability to COVID-19 Complications
Source: JAMA Netw Open. 2023 Oct 2;6(10):e2336678. doi: 10.1001/jamanetworkopen.2023.36678 (PMC10546233; doi:10.1001/jamanetworkopen.2023.36678)
Supplement: Supplement 2. — Data Sharing Statement [file jamanetwopen-e2336678-s002.pdf]

Data Sharing Statement

Dormuth. Nirmatrelvir-Ritonavir and COVID-19 Mortality and Hospitalization Among Patients With Vulnerability to COVID-19 Complications. *JAMA Netw Open*. Published October 02, 2023. doi:10.1001/jamanetworkopen.2023.36678

Data
Data available: No

Additional Information
Explanation for why data not available: The authors are not permitted to share the data used in this analysis. Individuals wishing to access the same source data should submit an application to Population Data BC (www.popdata.bc.ca), which administers access to provincial data for research purposes.
